# Spontaneous Intracranial Hypotension and Dural Ectasia in Marfan Syndrome: An Illustrative Case Successfully Treated with Steroid Therapy and Literature Review

**DOI:** 10.3390/brainsci14111143

**Published:** 2024-11-15

**Authors:** Francesco Signorelli, Omar Ktari, Ludovico Agostini, Giorgio Ducoli, Fabio Zeoli, Massimiliano Visocchi

**Affiliations:** 1 Institute of Neurosurgery, Fondazione Policlinico Universitario A. Gemelli IRCCS, Catholic University, 00168 Roma, Italy; ktariomar@gmail.com (O.K.); ludoago16@gmail.com (L.A.); ducoligiorgio@gmail.com (G.D.); fabio.zeoli01@icatt.it (F.Z.); mvisocchi@hotmail.com (M.V.); 2 Research Center and Master II Degree Surgical Approaches Craniovertebral Junction, Fondazione Policlinico Universitario A. Gemelli IRCCS, Catholic University, 00168 Roma, Italy

**Keywords:** spontaneous intracranial hypotension, Marfan Syndrome, epidural blood patch, steroid therapy

## Abstract

Background: Spontaneous intracranial hypotension (SIH) is a rare and frequently misdiagnosed disorder characterized by a low volume of cerebrospinal fluid (CSF) caused by the leakage of CSF through the spinal dural membrane. Patients with Marfan Syndrome (MS) and other connective tissue disorders are at an increased risk for dural ectasia, which may predispose them to spontaneous CSF leaks due to the structural weakness of their dural membranes. The management of SIH in MS patients is debated. Conservative measures, an epidural blood patch (EBP), and surgical treatments are the options generally provided. Methods: Herein, we report on the case of a 52-year-old female affected by MS, genetically confirmed, with a two-month history of sudden-onset, “thunderclap” headache, worsened in an upright position and horizontal diplopia. A Computed Tomography (CT) scan of the brain showed a bilateral chronic subdural hematoma, slit ventricles, and a caudal descent of the brainstem without overt tonsillar herniation. The Magnetic Resonance Imaging (MRI) scan of the whole spine revealed dural ectasia in the lumbosacral area and presacral perineural cyst without extradural CSF collection. The case was successfully managed with bed rest and high-dose corticosteroid therapy. Then, we discuss the pertinent literature, consisting of 25 papers dealing with the treatment of SIH in patients affected by MS. Results: The literature review yielded 25 papers dealing with SIH management in patients with MS, including 28 patients overall; 21 patients underwent EBP, of whom 7 patients had multiple procedures. Overall, in 23 cases (82%), the symptoms improved. In three cases, the patients were managed conservatively with bed rest. In three of these cases, there was an improvement. In one case, the surgical fenestration of two lumbar intradural spinal meningeal cysts was performed and the patient improved after the procedure. Our patient underwent 15 days of steroid therapy (dexamethasone iv 12 mg/day for 7 days, then reduced to 4 mg/day) and intravenous hydration (Ringer lactate 1500 mL/day). In ten days, the symptoms disappeared. At the 6-month follow-up, the patient was in good clinical condition, and a CT scan showed an almost complete regression of the bilateral subdural hematoma. Conclusions: The management of SIH in MS patients is still challenging. Patients with connective tissue disorders such as MS are at an increased risk for SIH. Few studies have assessed the management of these patients and different strategies. Our case and the available literature provide further data for this type of case.

## 1. Introduction

Spontaneous intracranial hypotension (SIH) is a condition of low cerebrospinal fluid (CSF) volume secondary to CSF leakage through a dural defect. The estimated incidence is 5 per 100,000 per year [1]. Orthostatic headache is the main symptom and bilateral subdural hygromas or subdural hematomas (SDHs) are common findings. Other symptoms may include nausea, vomiting, disorientation, the impairment of memory, diplopia, gait disturbances, cranial nerve palsies, sinus thrombosis, or coma [2,3,4]. Patients affected by connective tissue disorders, such as Marfan Syndrome (MS), Ehlers–Danlos syndrome type 2, and autosomal dominant polycystic kidney disease, are at an increased risk of SIH because of an intrinsic dural weakness which predisposes them to single or multiple CSF leaks. Moreover, the patient’s thin, dilated dura can be more permeable to CSF leakage, even without an overt laceration. The subsequent slow dilation of the sac can cause bone erosion and remodeling, thus leading to the widening of the vertebral canal [5,6]. Dural ectasia (DE), typically associated with collagenopathies, is an important diagnostic criterion of MS [7].

In some cases, SIH may resolve spontaneously without specific treatment. Initial conservative measures rely on bed rest and oral hydration. Epidural blood patching (EBP) is the most commonly performed intervention for spinal CSF leaks. It is a minimally invasive procedure in which a small volume of autologous blood is injected into the epidural space to seal the underlying CSF leak. In rare refractory instances, if the CSF leak has been definitively localized, surgical exploration may be an option [8].

The use of caffeine and theophylline and the abdominal binder are also reported in literature, but their effectiveness is not clear [8].

The use of steroids as part of the conservative treatment of SIH is debated, reflecting the fact that pathophysiological mechanisms underlying the potential efficacy of corticosteroids in SIH are not fully elucidated. It has been hypothesized that the benefits of steroids may result from a combination of fluid retention, the decrease in CSF hyperabsorption, and the enhanced CSF reabsorption from the extradural space, thus increasing its volume [9]. The possible role of steroid therapy in patients affected by MS and SIH with DE has not been explored so far.

Herein, we report on the case of a 52-year-old female affected by MS, presenting with symptomatic SIH secondary to DE, successfully managed with bed rest and high doses of corticosteroid therapy, and discuss the pertinent literature.

## 2. Materials and Methods

A review of the current literature was performed to identify original articles describing treatment of SIH in patients affected by MS. PubMed was searched for original studies using the search query “(Spontaneous Intracranial Hypotension) AND (Marfan Syndrome)” which yielded 42 results. No duplicate results were present in this search and six additional articles were identified through forward research, with 48 results overall. Abstract screening led to the exclusion of 23 results. The remaining 25 articles were included in this review.

## 3. Results

### 3.1. Case Presentation

**Figure 1:**
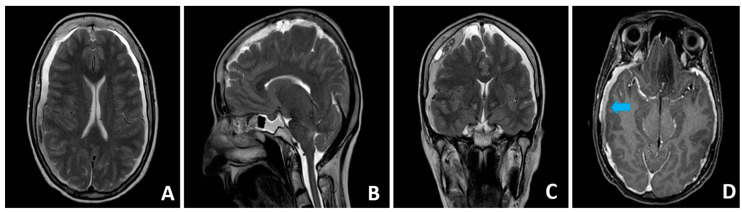
The MRI scan of the brain showed typical findings of intracranial hypotension. Axial and coronal T2-weighted images showing bilateral subdural fluid collections and reduction in size of the ventricular system (**A**,**C**). Sagittal T2-weighted image showing caudal descent of the brainstem and third ventricle, with minimal tonsillar herniation through the foramen magnum (**B**). Axial T1-weighted image after intravenous administration of gadolinium showing characteristic dural enhancement (**D**). Blue arrow: maximum thickness of subdural hematoma.

**Figure 2:**
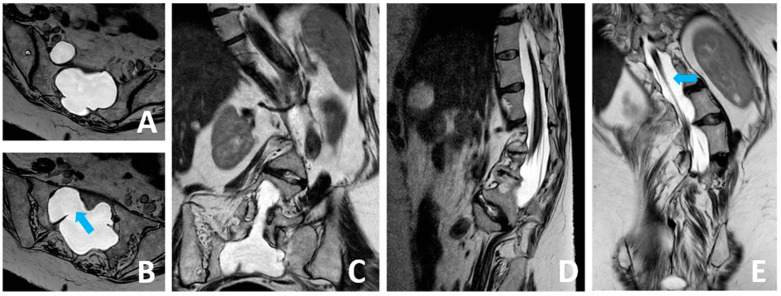
The MRI scan of the whole spine revealed dural ectesia (dAPn 60 mm × dLL 70 mm) in the lumbo-sacral area (**A**–**D**). Coronal T2-weighted image showing distal tract of the roots of the cauda equina laterally dislocated (**E**). The «mega-sac» appeared to be divided and isolated from remaining intrathecal compartment by arachnoid sepiments, as showed in axial T2-weighted images (**B**), thus resembling a «pseudocystic» dilatation. Blue arrow: arachnoid sepiments in axial (**B**) and sagittal (**E**) plane.

The patient, a 52-year-old female with a genetically confirmed diagnosis of MS determining severe thoracolumbar scoliosis, and aortic valve dysfunction requiring mechanical valve replacement on Warfarin anticoagulation, came to our attention after a two-month history of sudden-onset, “thunderclap” headache, which worsened in upright position and horizontal diplopia. No recent traumatic events were reported. A CT scan of the brain showed a bilateral hemispheric chronic subdural hematoma; slit ventricles were evidenced, along with caudal descent of the brainstem and the third ventricle, without tonsillar herniation. The MRI scan of the brain and the whole spine revealed dural ectasia (dAP 60 mm × dLL 70 mm) in the lumbosacral area, distal tract of the roots of the cauda equina laterally dislocated, and presence of presacral perineural cyst (Tarlov cyst), without overt CSF leakage. The “mega-sac” appeared to be divided and isolated from the remaining intrathecal compartment by arachnoid sepiments, thus resembling a “pseudocystic” dilatation (Figure 1 and Figure 2).

**Figure 3:**
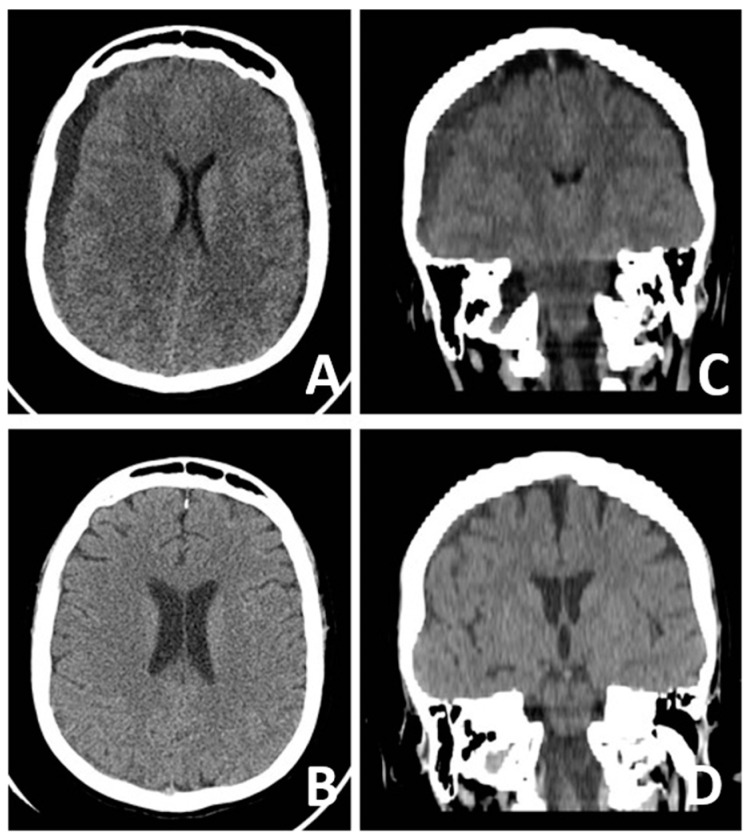
(**A**,**C**): Axial and coronal head CT scan images, acquired at admission, showing bilateral hemispheric subdural collections, the thickest of which are on the right, with minimum mass effect. (**B**,**D**): Axial and coronal head CT scan images, acquired one month after the patient was discharged, showing the almost complete resolution of the bilateral hemispheric subdural collections.

Physical examination revealed high-arched palate, mild pectus excavatum, arachnodactyly, long and thin arms, joint hypermobility, and bilateral flat foot. Coagulation tests showed an INR of 3.84, and Warfarin anticoagulation was discontinued and replaced by subcutaneous heparin the following day. During the first days, symptoms worsened and EBP was considered. However, the thrombotic risk associated with anticoagulation therapy (necessary in order to safely perform the procedure) discontinuation, the severe scoliosis, and the concomitant dural ectasia overall deterred us from performing the procedure, and, despite the initial neurological worsening, a conservative management was undertaken. The patient underwent 15 days of steroid therapy (dexamethasone iv 12 mg/day for 7 days, then reduced to 4 mg/day) and intravenous hydration (Ringer lactate 1500 mL/day). Then, neurological status improved rapidly, and, in ten days, symptoms disappeared. Gradual mobilization was well-tolerated. After two weeks, she was discharged home. At 6-month follow-up, patient was in good clinical condition and CT scan showed an almost complete regression of bilateral subdural hematoma (Figure 3).

### 3.2. Literature Search Overview

In total, 25 papers dealt with SIH management in patients with MS, including 28 patients overall; 21 patients underwent EBP, of whom 7 patients had multiple procedures (Table 1) [10,11,12,13,14,15,16,17,18,19,20,21,22,23,24,25,26,27,28,29,30,31,32,33,34]. Overall, in 23 cases (82%), symptoms improved. In four cases, patients were managed conservatively with bed rest. In three of these cases, there was an improvement. In one case, surgical fenestration of two lumbar intradural spinal meningeal cysts was performed, and the patient improved after the procedure.

## 4. Discussion

SIH is a rare but well-known condition, whose pathophysiology remains partly unclear. Despite the universally accepted diagnostic criteria recommended by the Headache Classification Committee of the International Headache Society [34], a great debate still exists concerning how to reliably diagnose and treat this condition, and the clinical results are often contradictory.

The management of suspected SIH begins with conservative measures before considering more invasive modalities. Initially, patients are hydrated and maintained in a flat position to reduce hydrostatic pressure and promote dural scaring [27]. Patients who do not improve or who deteriorate during observation may undergo EBP. Although this procedure has been used for many decades for SIH treatment, no randomized controlled studies have been performed to date. If patients continue to deteriorate or fail to improve following EBP, surgical repair of the dura should be considered for definitive therapy, whenever the source of the CSF leak is identified. Our group recently performed a systematic review and meta-analysis of the existing literature to evaluate the role of different factors possibly affecting the efficacy of the EBP procedure, by analyzing comparative studies reporting a clear description of patients experiencing good and poor responses to EBP [35].

DE occurs frequently in MS and, usually, it is asymptomatic. It occurs in the lower lumbar and sacral spine, with the thinning of the pedicles and the lamina, neural foraminal widening, or the presence of an anterior meningocele. In some cases, neurological symptoms can coexist, including headache, low back pain, and lower-limb paresthesia, which worsened in the orthostatic position. Despite DE representing a common feature of MS, its role in SIH has not been fully elucidated, especially in the case with no CSF leak.

It has been postulated that the pulsatile dynamics of CSF pressure in addition to minor asymptomatic trauma may lead to structural defects within the inherently weakened dura, thus predisposing patients to spontaneous CSF leaks [21].

In our case, the patient’s recent onset of symptoms along with evidence of a bilateral subdural hematoma without previous traumatic injury has oriented us towards a diagnosis of SIH in the context of DE, despite the absence of an overt CSF leak, suggesting an underlying valve CSF entrapment mechanism. Most likely, as described by Voermans et al. [23], the subarachnoid connections between the intradural pseudocystic ectasic mega-sac and the rest of the dural sac act as slit valves, opening synchronously with pulsation and rapidly closing. The subarachnoid connections along with the presacral perineural cyst may further enhance the phenomenon, resulting in spinal CSF steal and subsequently lowered CSF pressure above the pseudocystic mega-sac, and, finally, in postural headache.

Recently, in a meta-analysis of 144 articles dealing with possible treatments of SIH, D’Antona et al. [2] reported a successful conservative treatment in 28% of cases, mainly consisting of bed rest and hydration. The impact of other types of conservative treatment on the success rate was notably lower. The use of steroids accounted for only 4% (N = 30/748) of patients treated conservatively, probably as a result of the cut-off of at least 10 patients for study eligibility. This arbitrary threshold excluded small case series and case reports reporting the clinical improvement of SIH after steroid treatment [36,37].

The pathophysiological mechanisms underlying the potential efficacy of corticosteroids in SIH are still a matter of debate, mainly consisting in speculations without supporting data. Previous experimental evidence has demonstrated that steroids do not affect CSF production [38]. Goto recently reviewed the existing literature consisting of 18 cases of SIH treated with steroids [36] showed that the beneficial effects of steroids manifested anywhere between 1 day and several weeks, suggesting the involvement of different mechanisms. Steroids may enhance fluid retention, decreasing the inflammation of the meninges, decreasing the inflammatory response to the presence of cells or proteins in the CSF, or decreasing the vascular leakage. Moreover, steroids may repress CSF hyperabsorption and exert a reabsorption of the CSF from the extradural space [9].

Due to their anti-angiogenetic and anti-inflammatory effects, steroids have emerged as a non-surgical treatment option for chronic subdural hematomas [39], which are frequently observed in SIH.

Different types (dexamethasone, prednisone, methyl-prednisolone, or fludrocortisone), methods of administration (oral, intra-muscular, or intravenous), dosages of steroids, and durations of therapy have been described in the literature.

Dexamethasone is a long-acting glucocorticoid with plasma and biologic half-lives of 200 min and 36–54 h, respectively [40]. It is one of the most potent anti-inflammatory drugs but it lacks mineralocorticoid properties. Prednisolone has an intermediate duration of action with plasma and biologic half-lives of 115–200 min and 18–36 h, respectively [40]. Hydrocortisone is a corticosteroid, acting specifically as both a glucocorticoid and as a mineralocorticoid. Compared to hydrocortisone, prednisolone is about four times as potent and dexamethasone about 40 times as potent in terms of the anti-inflammatory effect. Hydrocortisone has been shown to be a safe and viable treatment option for patients with traumatic brain injury [39] by reducing hyponatremia due to its mineralocorticoid properties [41]. Recent evidence has shown that mild hypernatremia can decrease intracranial pressure, improving the cerebral perfusion pressure [42].

Adverse effects such as ecchymosis, cushingoid features, leg edema, and sleep disturbance are dose- and time-dependent. Other events (hypertension, glaucoma, weight gain, epistaxis, and depression) occur in the case of more prolonged treatments [10].

In the case of the MS patient who underwent corticosteroid administration as the first-line treatment [29], the steroid therapy was ineffective and EBP was performed, with no clinical improvement. Ten weeks following the onset of headache, the patient underwent a 7-day course of high-dose oral prednisone, followed by indomethacin 50 mg. Three days after beginning indomethacin, the headache disappeared. The authors conclude that, due to the nature of this connective tissue anomaly, the problem commonly recurs despite the type of treatment adopted.

In our case, the patient underwent 15 days of steroid therapy (dexamethasone iv 12 mg/die for 7 days, then reduced to 4 mg/day) and intravenous hydration (Ringer lactate 1500 mL/day). Then, she improved rapidly, and, in ten days, both the headache and horizontal diplopia disappeared.

The indication and results of EBP in patients with MS are also sparse and contradictory, mainly consisting in isolated case reports (see Table 1). Diaz et al. [13] reported a case of an adolescent female patient with SIH associated with multiple Tarlov cysts on an MRI, with no specific CSF leak sites on the radionuclide cisternography, who did not improve, despite four blood patch procedures. The authors point out the risk of producing more dural leaks by repeated unsuccessful lumbar EBPs, thus further complicating postural headache therapy in a patient with multiple Tarlov cysts and no CSF leak.

Davenport et al. [10] showed only a partial benefit from the blood patch procedure. They published the first reported case of SIH in MS. The authors postulate that the leak was caused by minor, unrecognied trauma rupturing one or more pre-existing spinal arachnoid diverticula, complicating the syndrome. They conclude that targeted EBP is the best treatment in the presence of severe symptoms. Bassani et al. [27] described a 58-year-old symptomatic woman with MS and mechanical aortic valve replacement on warfarin anticoagulation with the brain MRI showing the inferior displacement of cerebellar tonsils below the foramen magnum. Warfarin anticoagulation was stopped and replaced by heparin. Due to the risk associated with anticoagulation, the patient was considered ineligible for EBP and a conservative management consisting of bed rest and fluid therapy was undertaken. On the spinal MRI, DE was noted at multiple levels from L3-S2 and multiple sacral and thoracic root sleeve cysts and paraspinal thin bands of CSF were observed in the left thoracic region, consistent with CSF extravasation. Those anatomical conditions and the concomitant anticoagulation would cause the lumbar puncture and EBP procedure to be at high risk for possible thoracolumbar epidural hematomas. This case is very similar to ours and share comparable controversies. Indeed, in our case, the patient has presented with more severe and debilitating clinical conditions due to the persistence of headache and diplopia despite the initial bed rest and hydration. Furthermore, our patient had a pronounced severe scoliosis such as to make the lumbar puncture technically challenging and hazardous. The severe clinical picture, along with the impossibility of proceeding with EBP, prompted us to maximize the conservative management with steroid treatment.

The disappearance of the epidural fluid collection sometimes observed after steroid treatment may suggest that the anti-inflammatory effect of steroid can promote dural repair [36]. This observation may be even more appropriate in conditions of intrinsic dural fragility such as MS. The specific role of steroid therapy in MS patients harboring SIH with DE and no CSF leakage has not been explored. It can be postulated that the enhanced fluid retention guaranteed by steroids can reverse the aspiration force in the context of the ectasic mega-sac.

## 5. Conclusions

Both congenital and acquired factors make SIH a concrete and probably underestimated entity in MS patients, whose management still remains challenging. According to the literature, EBP remains the most successful treatment of SIH. Nevertheless, specific anatomical features and the intrinsic systemic fragility of this type of patient can make EBP risky as well as ineffective, especially when the CSF leak is missing, paradoxically exacerbating postural headache.

Our case raises several considerations surrounding the frequent features of SIH in patients affected by MS. In particular, we highlight the pivotal role of conservative management in patients with multiple comorbidities needing long-term anticoagulation, for whom the EBP procedure is fraught with excessive risks. Steroid therapy, effective in our case, has not been carried out or has not brought any appreciable benefits in similar reported cases, further demonstrating that the possible underlying mechanism of steroid-mediated dural repair have not yet been understood. Further studies are needed to demonstrate the effectiveness of conservative management and steroid therapy in this subset of patients.

## Figures and Tables

**Table 1:** SIH management in patients with MS. DE: dural ectasia; NRE: nerve root ectasia; TCy: Tarlow cyst; EBP: epidural blood patch.

Authors	N pts.	Sex	Age (Y)	CSF-Leak	Dural Features	Treatment	F Up (m)	Outcome
Davenport, 1995 [10]	1	F	19	-	NRE	2 EBP	-	Improv.
Fukutake et al., 1997 [11]	1	F	30	yes	-	1 EBP (blind)	-	Improv.
Deputy et al., 1999 [12]	1	F	15	no	DE	2 EBP (blind)	-	No improv.
Diaz et al., 2001 [13]	1	F	16	no	NRE	4 EBP (blind)	-	No improv.
Ferrante et al., 2003 [14]	1	M	26	–	DE, NRE	Conserv. manag. (bed rest)	3	Improv.
Owler et al., 2004 [15]	1	F	27	no	-	Conserv. manag. (bed rest)	4	Improv.
Milledge et al., 2005 [16]	1	F	14	no	DE, TCy	1 EBP (blind)	12	Improv.
Rosser et al., 2005 [17]	1	F	10	yes	NRE	1 EBP (lumbar)	4	Improv.
Puget et al., 2007 [18]	1	F	12	-	DE, NRE	1 EBP	15	Improv.
Albayram et al., 2008 [19]	1	F	32	yes	-	1 EBP (lumbar)	3	Improv.
Cheuret et al., 2008 [20]	1	F	14	no	DE, NRE	1 EBP (blind)	12	Improv.
Decontee et al., 2009 [21]	1	M	47	no	DE	Cerv., thorac., lumbar EBPHigh-dose oral prednisone		No improv.
Rosdy et al., 2009 [22]	1	F	15	yes	-	1 epid. saline injection (blind)	30	Improv.
Voermans et al., 2009 [23]	1	M	13	-	DE, NRE, TCy	Surgery: cyst fenestration	-	Improv.
Pabaney et al., 2010 [24]	1	M	38	yes	NRE	1 EBP (blind)	6	Improv.
Khalid et al., 2012 [25]	1	M	38	-	-	1 EBP (blind)	-	Improv.
Schievink et al., 2013 [26]	3	2F M	18, 13 19	- -	3 DE	2 EBP 1 surgery	-	Improv. (1 pt died 18 m after surgery for aortic dissection)
Bassani et al., 2014 [27]	1	F	58	yes	DE, NRE	Conserv. manag. (bed rest)	15	Improv.
Abu Libdeh et al., 2016 [28]	1	M	18		-	Conserve. manag. (bed rest)	-	-
Apetroae et al., 2016 [29]	2	2F	22, 27	- yes - no	NRE	2 EBP (blind) 1 EBP (blind)	10 6	Improv.
Mirchi et al., 2020 [30]	1	M	12	yes	NRE	Multilevel EBP	-	-
Pichott et al., 2020 [5]	1	F	13	no	DE	2 EBP (blind)	-	Improv.
Cerulli et al., 2021 [31]	1	F	42	-	-	1 EBP	2	Improv.
Sanchez et al., 2023 [32]	1	M	11	yes	DE	1 EBP	12	Improv.
Tariq et al., 2024 [33]	1	F	18	yes	NRE	2 EBP (blind)	-	Improv.
Present Case	1	F	52	no		Dexamethasone	12	Improv.

## Data Availability

All the data generated in the present study have been included in this paper after anonymization.
